# Role of C-Reactive Protein in Predicting the Severity and Response of Immune-Mediated Diarrhea and Colitis in Patients with Cancer

**DOI:** 10.7150/jca.84261

**Published:** 2023-06-26

**Authors:** Cynthia Liu, Malek Shatila, Antony Mathew, Antonio Pizuorno Machado, Austin Thomas, Hao Chi Zhang, Anusha S. Thomas, David Faleck, Pauline Funchain, Jessica Philpott, Petros Grivas, Michel Obeid, Franck Carbonnel, Yinghong Wang

**Affiliations:** 1 Department of Internal Medicine, Baylor College of Medicine, Houston, TX, USA.; 2 Department of Gastroenterology, Hepatology and Nutrition, The University of Texas MD Anderson Cancer Center, Houston, TX, USA.; 3 Department of Internal Medicine, The University of Texas Health Science Center, Houston, TX, USA.; 4 Gastroenterology, Hepatology and Nutrition Service, Department of Medicine, Memorial Sloan Kettering Cancer Center, New York, NY, USA.; 5 Department of Hematology and Medical Oncology, Cleveland Clinic, Cleveland, OH, USA.; 6 Center for Inflammatory Bowel Disease, Cleveland Clinic, Cleveland, OH, USA.; 7 Department of Medicine, Division of Oncology, University of Washington, Clinical Research Division, Fred Hutchinson Cancer Center, Seattle, WA, USA.; 8 Centre Hospitalier Universitaire Vaudois, Department of Medicine, Service of Immunology and Allergy, Lausanne, Switzerland.; 9 Gastroenterology Department, Université Paris Saclay 11, Le Kremlin-Bicêtre, France.

**Keywords:** immunotherapy, immune-mediated, diarrhea, colitis, C-reactive protein, fecal calprotectin

## Abstract

**Background:** Immune-mediated diarrhea and colitis (IMDC) frequently develop after treatment with immune checkpoint inhibitors. C-reactive protein (CRP) is a serum inflammatory biomarker used to stratify and monitor disease severity in many inflammatory conditions. However, CRP level is not specific and is widely influenced by various factors non-specific to bowel inflammation. We aimed to study the utility of CRP as a predictor of disease severity and therapy response in IMDC.

**Methods:** We performed a retrospective analysis of patients diagnosed with IMDC who had CRP measured at IMDC onset and after treatment with selective immunosuppressive therapy (SIT: infliximab and vedolizumab), between 01/2016 and 02/2022 at MD Anderson Cancer Center. Patient demographics, clinical characteristics, and IMDC data were collected and analyzed.

**Results:** Our sample of 128 patients had a median age of 67 years; most were white (89.8%); and male (65.6%). Prior to development of IMDC, 15 (11.7%) were initially treated with anti-CTLA-4, 42 (32.8%) with anti-PD-1 or PD-L1, and 71 (55.5%) with a combination of both. We found higher CRP level was associated with higher CTCAE grade of clinical symptoms such as diarrhea (p=0.015), colitis (p=0.013), and endoscopic findings (p=0.016). While CRP levels decreased after IMDC treatment, there was no significant association between CRP levels with clinical remission, endoscopic remission or histologic remission. There also was no significant correlation between CRP level and recurrence of IMDC, or with fecal calprotectin levels.

**Conclusion:** CRP level may be useful to assess initial severity of IMDC, including grade of diarrhea and colitis and degree of endoscopic inflammation. However, CRP is not a robust surrogate biomarker for assessing treatment response or disease recurrence. Despite the reduction of CRP levels observed following IMDC treatment, this finding might be nonspecific and potentially confounded by concurrent clinical factors, such as underlying malignancy, other inflammatory processes, and systemic anti-cancer therapy. Further studies of the role of CRP are warranted in patients with cancer and IMDC.

## Introduction

Biomarkers are used in inflammatory diseases to assess inflammatory burden, monitor treatment response, and assess risk of relapse. Ideal biomarkers would be expected to be easy to use, sensitive, and specific to an active inflammatory state, with rapid turnaround time and low cost. C-reactive protein (CRP) is a well-known acute-phase reactant commonly used as a biomarker of inflammation in many inflammatory conditions but has been shown to be widely influenced by other inflammatory and infectious states and are nonspecific to bowel inflammation (autoimmune disease, cancer, sepsis, etc). CRP has been used anecdotally as surrogate marker for disease activity in immune mediated diarrhea and colitis (IMDC), which is an entity that arises as an immune checkpoint inhibitor (ICI) related adverse effect. As the scope of treatment of ICIs has expanded across many cancer types, IMDC has become a more prevalent diagnosis. Though CRP has been studied as a biomarker in other inflammatory conditions, it has not been widely studied in the context of IMDC.

Studies suggest that an estimated 15% of patients lack the ability to mount an elevated quantity of CRP due to genetic variations 1, 2. Its use, though nonspecific, has been shown to correlate with severity of inflammatory bowel diseases (IBD) as assessed endoscopically 3. A meta-analysis suggested that CRP has a relatively high specificity (92%), but lower sensitivity (49%), to predict endoscopic activity in patients with known IBD, and normal CRP level does not exclude the possibility of active disease 4. Fecal calprotectin (FCal) is a protein marker shed in stool that produced as a result of neutrophil migration to the intestinal mucosa that is both more sensitive and more specific than CRP as a biomarker in the course of IBD 5.

Although these biomarkers are well studied in relation to IBD, less is known of their sensitivity, specificity, and thus clinical utility in the disease course of immune checkpoint inhibitor (ICI)-mediated diarrhea and colitis (IMDC) in the context of malignancy. In advanced non-small cell lung cancer, a potential correlation between CRP elevation and poorer cancer response to ICI therapy, in addition to the correlation between an early decline in CRP and better response to ICI therapy, has been demonstrated 6. Other studies have found a correlation between CRP elevation and the early detection of immune-related adverse events (irAEs) in melanomas treated with ICIs 7, 8. A recent case series suggested a strong correlation between FCal level and severity of ICI-related colitis on endoscopic exam 9. However, to our knowledge, only one study has explored the utility of CRP as a biomarker in IMDC 10. This study found no association between point-CRP and IMDC clinical or endoscopic outcomes but failed to explore the impact of changes to CRP level across the disease course. Additionally, CRP is an inexpensive test with results quickly available 11, as opposed to FCal which takes significantly longer 12. For this reason, it is important to explore the potential of CRP in this disease entity.

We aimed to study the correlation between CRP level and IMDC clinical outcomes, as well as the correlation between CRP and FCal levels. We predicted that CRP would have a poor correlation with endoscopic findings, disease course, and response to treatment in IMDC due to its nonspecific nature, as well as poor correlation with FCal, which is known to be a more specific biomarker in IMDC.

## Materials and Methods

### Patient Selection

This study, performed at a tertiary cancer center, was a retrospective analysis of adult patients with a cancer diagnosis who received ICI therapy between 01/2016 and 02/2022. We included patients with any malignancy type who received ICI, developed IMDC, and had CRP levels measured both at the time of IMDC diagnosis and after selective immunosuppressant treatment (SIT: infliximab and vedolizumab). Patients who did not have any CRP measurements or those with CRP-levels obtained for infectious causes were excluded. Patients with known active gastrointestinal infection at the time of IMDC diagnosis were also excluded. Approval was obtained from the Institutional Review Board (PA18-0472). Informed consent was waived due to the retrospective nature of this study.

### Clinical Data Collection

All clinical data were extracted from the institutional electronic medical record (EMR) and pharmacy databases. Patient demographic data; type and stage of cancer; cancer treatments; mortality; IMDC diagnosis, treatment, and complications; and CRP and FCal measurements were extracted from the EMR. The severity of diarrhea and colitis were assessed using the Common Terminology Criteria for Adverse Events, version 5. We collected data on CRP levels at the time of ICI initiation, at IMDC onset, at SIT initiation, peak levels during the IMDC course, and the latest level after medical treatment as a post-treatment value. Data from endoscopic findings at the time of diagnosis and subsequent endoscopies were collected. Endoscopic findings were characterized as ulcers, nonulcerative inflammation (e.g., erythema, friability, erosions, inflammatory exudate, loss of vascular pattern), or normal. The histologic findings were described as normal, acute active colitis (e.g., cryptitis, crypt abscess, apoptosis, eosinophilic infiltration, intraepithelial neutrophil infiltration), chronic active colitis (e.g., crypt architectural distortion, basal lymphoplasmacytosis, Paneth cell metaplasia), or microscopic colitis (e.g., intraepithelial lymphocytic infiltration, subepithelial collagen bands). The endoscopic and histologic categorizations were based on our prior published study on the endoscopic evaluation of ICI-induced colitis 8.

### Clinical Outcomes Assessment

Clinical remission of IMDC was defined as resolution of gastrointestinal symptoms (CTCAE grade ≤1) during the study window, and response was defined as improvement in the CTCAE severity of gastrointestinal symptoms (a decrease in CTCAE grade but still >1). Endoscopic and histologic remission were defined as having normal gross and histologic findings after treatment, with previously abnormal examinations. The follow-up duration was measured from the onset of IMDC to the date of death or last follow-up examination.

### Statistical Analysis

The distribution of each continuous variable was summarized by its median and interquartile range. The distribution of each categorical variable was summarized in terms of its frequency and percentage. Independent sample and paired-sample *t*-tests and Mann-Whitney U test were used to compare the CRP levels between different groups after testing for normality. Finally, univariate logistic regression was used to test the association between CRP levels and different types of remission (i.e., clinical, endoscopic, and histologic) and recurrence. To examine the strength of association between CRP and FCal, Pearson correlation analysis was used. All tests were two-sided, and p-values less than 0.05 were considered significant. The computations were carried out using SPSS version 26 statistical software.

## Results

### Baseline Patient Characteristics

Our analysis included 128 patients (**Suppl. Figure 1**, patient selection flowchart), whose baseline demographics are summarized in **Table 1**. The median age at IMDC onset was 67 years. Most patients were male (65.6%), and white race (89.8%). Cancer type included genitourinary cancer (37.5%), melanoma (31.3%), lung cancer (13.2%), and others (18.0%). All patients had advanced cancer (stage IV, 88.1%). Most patients received a combination of both CTLA-4 and PD-(L)1 checkpoint inhibitors (55.5%), while others received either CTLA-4 (11.7%) or PD-(L)1 monotherapy (32.8%). Patients were followed up for a median of 48.5 months, and the all-cause mortality rate was 38.6%. Thirty-two patients developed other irAEs during the course of their colitis.

### CTCAE severity of IMDC Related to CRP Level

The median baseline CRP level at ICI initiation was 8.9 (1.0-30.2) compared to 21.6 (8.1-50.5) at IMDC onset (p=0.169). The association between CTCAE grade and CRP were analyzed and are shown in **Table 2**. The median baseline CRP levels after IMDC diagnosis were 12.1 mg/L in patients with grade 1-2 diarrhea and 35.1 mg/L in patients with grade 3-4 diarrhea (p=0.015). The median baseline CRP levels were 17.1 mg/L in patients with grade 1 colitis and 49.0 mg/L in patients with grade 2-4 colitis (p=0.013). CRP levels dropped after medical treatment in each group; however, no significant difference was observed between post-treatment CRP levels based on grade of symptoms. Patients with active inflammation seen on endoscopic evaluation, including ulcer or non-ulcer inflammation, had a median baseline CRP level of 30.1 mg/L, while those with normal endoscopic findings had a baseline CRP level of 15.4 mg/L (p=0.016). A consistent pattern of reduction in CRP level after treatment was demonstrated, with comparable values for patients with inflammation and with normal findings (p=0.397). No significant association was found between the peak CRP levels in the disease course or the change in CRP before and after medical treatment and different severity grades of CTCAE diarrhea or colitis; endoscopic inflammation; and the presence of other concurrent irAEs. There was no significant difference identified in the extent of CRP reduction based on SIT type (data not shown).

### IMDC Outcomes Related to CRP Level

**Figure 1:**
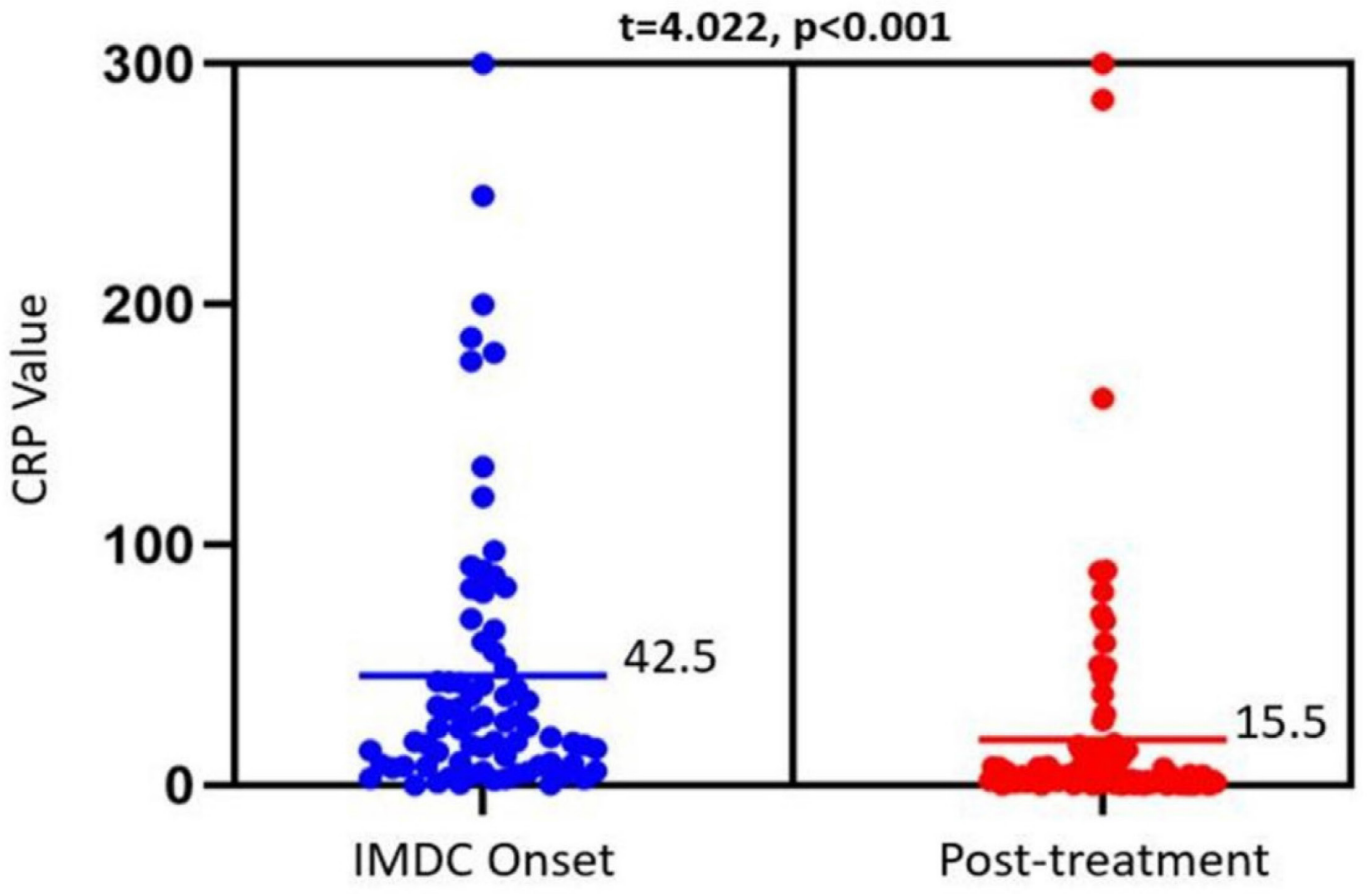
Dot plot showing CRP levels obtained at IMDC onset (left) and after treatment with SIT agents (right). The bars reflect the mean CRP values (mg/L) for each timepoint, which were compared via a paired *t*-test (results at the top). Mean CRP level was used for this figure for better illustration, with comparable p value as median in the tables.

IMDC outcomes and their association with CRP levels at IMDC onset and after SIT are summarized in **Table 3**. There was a significant reduction in CRP from 42.5 mg/L to 15.5 mg/L, p<0.001) after medical treatment (**Figure 1**). This trend was not observed among different IMDC outcomes, as shown in **Table 3**. Patients with clinical, endoscopic, or histologic remission had similar CRP levels pre- and post-treatment as those without remission. This was also true when looking at peak CRP levels or the delta in CRP levels pre- and post-treatment (**Table 4**). Finally, no difference was observed in pre- and post-treatment CRP levels between patients with and without IMDC recurrence.

### Correlation of CRP and FCal

CRP and FCal levels and their correlation at the onset of IMDC and after treatment are displayed in **Supplementary Table 1**. CRP level did not correlate with FCal level at IMDC onset (correlation coefficient 0.05, p=0.621), after medical treatment (correlation coefficient=0.12, p=0.313), or change in CRP levels (correlation coefficient 0.10, p=0.448).

## Discussion

Serum CRP level is used as a biomarker in inflammatory conditions such as IBD and autoimmune disease but remains understudied in the realm of IMDC. Particularly, its correlation with severity and disease course or its correlation with FCal, another ubiquitous biomarker in IMDC, is not well-established. Our study demonstrated that CRP level at IMDC onset correlates with CTCAE grade of diarrhea and colitis, as well as degree of endoscopic inflammation. There was a significant reduction in CRP values after IMDC medical treatment compared with baseline; however, no significant relationship was found between the last recorded CRP level or change in CRP level across treatment and clinical, endoscopic, or histologic remission or recurrence of IMDC. Therefore, CRP may be useful as a baseline biomarker for disease severity but is not a reliable surrogate biomarker to predict remission. Furthermore, we found poor correlation with FCal levels for monitoring of IMDC disease status.

Serum CRP, unlike FCal, is an inflammatory biomarker that is nonspecific to bowel inflammation. It is an acute phase reactant that is produced by the liver when stimulated by cytokines interleukin-6, tumor necrosis factor (TNF)-α, and interleukin (IL)-1β in inflammatory states, whereas FCal is a biomarker for neutrophil migration to the bowel wall 13. When used as a surrogate biomarker in certain autoimmune conditions, CRP has been found to be less specific and useful than other biomarkers. For example, in systemic lupus erythematosus, biomarkers such as IL-18 and TNFα are preferred over CRP for disease monitoring 14. Similarly, in IMDC, FCal has been found to be a more specific biomarker for monitoring of colitis 15. CRP did not correlate to treatment response in terms of clinical, endoscopic or histologic remission or recurrence in our study. We suspect several confounding variables contributing to the limited utility for CRP in IMDC disease monitoring. For example, the patients with IMDC were all being treated with ICIs for underlying malignancy. Malignancy itself has been shown to cause elevations in CRP level 16. In addition, patients with underlying malignancy are often older with more co-morbid conditions, further confounding CRP elevations. The use of a corticosteroid as part of IMDC treatment and other irAEs related to ICI treatment could also affect CRP values although our study did not observe the association between CRP level and presence of other concurrent irAEs likely due to small sample size in this subgroup.

CRP does have utility for assessment of disease severity and progression in some conditions such as rheumatoid arthritis, malignancy, and IBD. In rheumatoid arthritis, higher CRP levels are associated with greater disease activity and radiological damage 17. However, the clinical utility has also been called into question; for example, a substantial number of patients with rheumatoid arthritis flares have normal CRP values. Future risk of developing cancer, as well as an increased risk of death in patients with active malignancy, was found to be associated with higher baseline CRP levels (>3 mg/L) compared to low levels (<1 mg/L) 16. Studies have shown that higher CRP-to-albumin ratios are associated with relatively worse prognosis from early-stage to metastatic cancers, and across all types, including genitourinary, gastrointestinal, lung, and breast cancers 18-23. Although CRP has been shown to have prognostic associations in these malignant conditions, the lack of specificity still presents a significant limiting factor for its clinical utility.

With regard to IBD, CRP level has also been found to be significantly associated with disease activity in Crohn's disease and ulcerative colitis. A prospective study by Henriksen et al. showed that CRP levels at diagnosis had correlation with extent of ulcerative colitis 24. CRP is also used to predict prognosis and relapse of Crohn's disease 10. Like with IBD, the CRP level on initial presentation of IMDC did correlate to initial disease severity and presence of endoscopic inflammation. However, the lack of correlation of CRP level to treatment response in IMDC makes it not useful as a biomarker to monitor disease progression. Given the role of CRP levels as both evaluative and prognostic biomarkers of IBD, this lab test has been readily adopted in the current practice of IMDC. While IMDC shares some features in common with IBD, IMDC is still a distinct entity, which may explain the observation of our study that CRP level is less informative in the context of more confounding factors present in a population of patients with cancer. Therefore, the clinical significance of CRP may be less broad for IMDC compared to its value in IBD, and more studies are needed to explore its role in this population.

Interestingly, Cheung et al. found lack of correlation between CRP level and IMDC severity, measured by need for steroids or infliximab, which is a different measure from our study that utilized CTCAE grade of diarrhea and colitis 10. However, one would possibly expect similar findings since the need for a steroid or infliximab is usually more frequent among patients with higher CTCAE grade of clinical symptoms. Current guidelines consider predominantly CTCAE grade of reported diarrhea and colitis symptoms for clinical management; thus, their correlation with CRP is relevant for initial evaluation of IMDC severity. Moreover, we found that there was a significant association between CRP value and endoscopic severity at IMDC onset, specifically when active inflammation was present on endoscopy, which serves as the gold standard for objective evidence of colitis. Prior studies have found correlation between FCal level and eventual endoscopic and histologic remission in IMDC, but CRP has not been independently studied 9. The lack of correlation between CRP and clinical or endoscopic remission after treatment that we observed was likely attributable to increased complexity of the clinical and biochemical factors throughout the IMDC disease course that may affect CRP values. On the other hand, FCal reflects the degree of colonic mucosal neutrophils and therefore increases specifically with colonic inflammation. FCal and CRP levels had poor correlation to one another both before and after IMDC treatment in our study. Overall, FCal appears to be a more reliable and informative lab parameter than CRP to check and monitor for IMDC.

Our study bears several limitations. First, it was a retrospective single-center study, which limited our data collection to the available information documented in the EMR. Second, we excluded patients who did not have CRP values recorded during the IMDC disease course, e.g., those who did not receive SIT, which could impact our sample size and may have introduced selection bias. We limited our study to patients treated with SITs since these patients were more likely to be followed closely and had CRP levels checked throughout disease course. Third, current clinical practice does not routinely check baseline CRP level before IMDC diagnosis, excluding the possibility of exploring the role of CRP at different stages of the cancer treatment course. Last, all of the study patients were being treated for active cancer; therefore, the respective CRP values could have been confounded by the cancer type and status, anti-cancer therapies, other medications, steroids, infection, and/or other active inflammatory states (such as other non-gastrointestinal adverse events related to ICI) that are unable to be addressed thoroughly in this study.

The initial serum CRP level in IMDC correlates with clinical colitis CTCAE severity, as well as initial endoscopic presentation, however, post-treatment CRP level has no correlation to IMDC remission or recurrence. CRP could be used during initial IMDC evaluation to stratify for severity but would serve as a suboptimal surrogate biomarker for monitoring IMDC disease course and outcome, in which case, FCal is a more reliable biomarker. Future studies should further evaluate the role of CRP in populations with cancer treated with ICIs as a potential guide for initial IMDC treatment.

## Supplementary Material

Supplementary figure and table.

## Figures and Tables

**Table 1:** Patients' baseline demographic and clinical characteristics (N=128)

Characteristic	No. of patients (%) or median (IQR)
Median age at IMDC (IQR) - yr, n=128	67 (58-73)
Sex	
Male - no. (%)	84 (65.6%)
Female- no. (%)	44(34.4%)
Race	
White race - no. (%)	115 (89.8%)
Other - no. (%)	13(10.2%)
Cancer type - no. (%), n = 128	
Genitourinary cancer	48 (37.5%)
Melanoma	40 (31.3%)
Lung cancer	17 (13.2%)
Others	23 (18.0%)
Cancer stage^1^ - no. (%), n=127^2^	
III	11 (8.7%)
IV	112 (88.1%)
Class of ICI - no. (%), n=128	
Anti-CTLA-4	15 (11.7%)
Anti-PD-(L)1	42 (32.8%)
Combination	71 (55.5%)
Other irAEs concurrent to IMDC^3^, n=123^2^	32 (26.0%)
Median follow-up duration (IQR) - mo., n=128	48.5 (26.3-81.1)
All-cause mortality	49 (38.6%)

IMDC, immune-mediated diarrhea and colitis; IQR, interquartile range; ICI, immune checkpoint inhibitor; CTLA-4, cytotoxic T lymphocyte antigen 4; PD-(L)1, programmed cell death 1/programmed cell death 1 ligand 1. ^1^4 Patients had stage 1 or 2 cancer. ^2^Missing info: Some information was not available in the electronic health record and therefore could not be included in the final descriptive analysis. ^3^Other non-gastrointestinal irAEs included: dermatologic (10), endocrine (8), musculoskeletal (7), hepatic (3), neurologic (2), renal (1), and pulmonary (1) toxicity.

**Table 2:** Association between IMDC severity and CRP levels before and after treatment

Characteristic	N	Initial CRP Median (IQR)	p value	N	Post-treatment CRP Median (IQR)	p value
Diarrhea grade			0.015			0.238
1-2	23	12.1 (5.3-31.4)		38	3.4 (1.0-7.6)	
3-4	43	35.1 (9.6-69.0)		61	4.1 (1.3-15.1)	
Colitis grade			0.013			0.183
1	19	17.1 (7.8-28.9)		23	2.6 (1.0-4.5)	
2-4	30	49.0 (12.1-82.4)		52	4.4 (1.9-14.9)	
Endoscopic presentation			0.016			0.397
Active inflammation (ulcer ± non-ulcer inflammation)	52	30.1 (9.1-72.4)		81	2.6 (1.0-9.1)	
Normal	16	15.4 (5.9-23.8)		21	3.8 (1.3-14.2)	

IMDC, immune-mediated diarrhea and colitis; CRP, C-reactive protein; IQR, interquartile range. Mann-Whitney U test was used for analysis and reported as median and IQR.

**Table 3:** Association between IMDC outcomes and CRP levels before and after treatment

Characteristic	N	Initial CRP Median (IQR)	p value	N	Post-treatment CRP Median (IQR)	p value
Clinical remission			0.146			0.680
Yes	54	27.5 (9.4-59.5)		84	3.5 (1.2-13.5)	
No	15	14.5 (7.8-32.3)		18	4.1 (1.0-10.7)	
Endoscopic remission			0.884			0.063
Yes	18	20.7 (8.6-59.5)		32	2.2 (0.6-7.2)	
No	12	20.0 (8.7-49.3)		23	4.0 (2.0-10.3)	
Histologic remission			0.613			0.143
Yes	14	18.1 (12.1-40.0)		24	2.0 (0.6-6.2)	
No	18	27.8 (8.6-69.0)		33	3.8 (1.9-9.1)	
Symptom recurrence			0.310			0.512
Yes	18	27.5 (15.2-55.4)		29	3.8(1.6-17.5)	
No	52	17.9 (7.4-45.8)		72	3.5(1.0-10.9)	

Footnote: Mann-Whitney U test was used and reported as median and IQR.

**Table 4:** Univariate logistic regression analysis of association of CRP levels with clinical (n=69), endoscopic (n=30), and histologic remission (n=32)

	Last CRP level after medical treatment		Delta in pre- and post-treatment CRP levels	
Outcome	OR (CI)	p value	OR (CI)	p value
Clinical remission	1.01 (1.0-1.01)	0.302	0.99 (0.99-1.00)	0.153
Endoscopic remission	0.99 (0.98-1.01)	0.421	1.00 (0.99-1.01)	0.896
Histologic remission	0.99 (0.97-1.02)	0.441	1.01 (1.00-1.02)	0.153

CRP, C-reactive protein; OR, odds ratio; CI, confidence interval.

## Abbreviations

CRP: C-reactive protein
CTCAE: Common Terminology Criteria for Adverse Events
CTLA-4: cytotoxic T-lymphocyte-associated protein 4
EMR: Electronic medical record
Fcal: fecal calprotectin
ICI: immune checkpoint inhibitor
IMDC: immune-mediated diarrhea and colitis
IQR: interquartile range
irAE: immune-related adverse event
PD-(L)1: programmed cell death protein 1 or programmed cell death 1 ligand 1
SEM: standard error of the mean
SIT: selective immunosuppressive treatment
