# Supplementary material for: Role of C-Reactive Protein in Predicting the Severity and Response of Immune-Mediated Diarrhea and Colitis in Patients with Cancer
Source: J Cancer. 2023 Jun 26;14(10):1913–9. doi: 10.7150/jca.84261 (PMC10355204; doi:10.7150/jca.84261)
Supplement: Supplementary file 1 — Supplementary figure and table. [file jcav14p1913s1.pdf]

Supplemental Table 1. Association between C-reactive protein and fecal calprotectin levels at different time points in IMDC disease course		
**CRP parameter**	**Correlation coefficient**	**p value**
Initial baseline CRP levels	0.05	0.621
Post-treatment CRP levels	0.12	0.313
Change in CRP levels	0.10	0.448
Pearson correlation was used for analysis.		

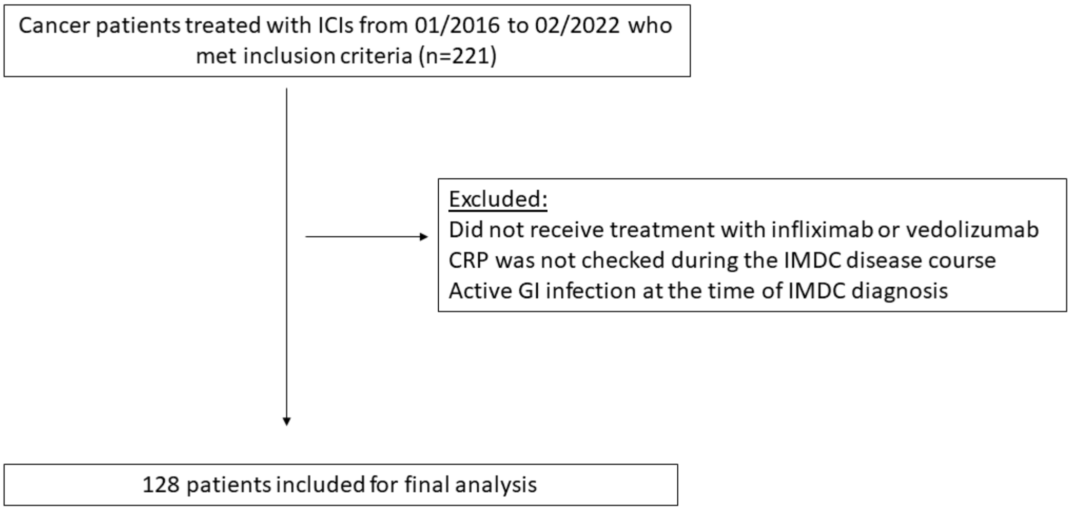

Supplementary Figure 1. Patient Selection Flowchart
